# Variables associated with endogenous hyperinsulinism in hypoglycemia diagnosis. Could the 72-hour fasting test be shortened in low-risk patients?

**DOI:** 10.1016/j.jcte.2025.100386

**Published:** 2025-03-07

**Authors:** Tomás González-Vidal, Óscar Lado-Baleato, Inés Masid, Carmen Gándara-Gutiérrez, Gema Martínez-Tamés, Jessica Ares, Carmen Lambert, María Riestra-Fernández, Francisco Gude, Elías Delgado, Edelmiro Menéndez-Torre

**Affiliations:** a Department of Endocrinology and Nutrition, Hospital Universitario Central de Asturias/University of Oviedo, Spain; b Department of Medicine, University of Oviedo, Spain; c Instituto de Investigación Sanitaria del Principado de Asturias (ISPA), Oviedo, Spain; d Research Methods Group (RESMET), Health Research Institute of Santiago de Compostela (IDIS), Spain; e ISCIII Support Platforms for Clinical Research, Health Research Institute of Santiago de Compostela, Spain; f Department of Endocrinology and Nutrition, Hospital Universitario de Cabueñes, Gijón, Spain; g Department of Endocrinology and Nutrition, Hospital Valle del Nalón, Langreo, Spain; h Department of Psychiatry, Radiology, Public Health, Nursing and Medicine, University of Santiago de Compostela, Spain; i Concepción Arenal Primary Care Center, Santiago de Compostela, Spain; j Centre for Biomedical Network Research on Rare Diseases (CIBERER), Instituto de Salud Carlos III, Madrid, Spain

**Keywords:** Hypoglycemia, Fasting test, Endogenous hyperinsulinism, Insulinoma

## Abstract

**Background:**

The 72-hour fasting test remains the standard for the diagnosis of endogenous hyperinsulinism. We investigated which variables could identify patients at low risk for endogenous hyperinsulinism, in whom a shortening of the 72-hour fasting test could be considered.

**Methods:**

This multicenter, retrospective study included 64 individuals (46 women, median age 45 years) without diabetes who underwent 72-hour fasting tests for the etiologic diagnosis of hypoglycemia. Pre- and intra-test variables were collected, including point-of-care glucose trajectories during the test. Testing was stopped before 72 h if symptomatic serum glucose <55 mg/dL or asymptomatic serum glucose ≤45 mg/dL occurred. Endogenous hyperinsulinism was diagnosed in individuals who had serum glucose <55 mg/dL, serum insulin ≥3.0 μU/mL, and serum C-peptide ≥0.6 ng/mL.

**Results:**

Patients with endogenous hyperinsulinism (n = 10) had steeper descending point-of-care glucose trajectories (p < 0.001) than those without it. Older age and lower minimum pre-test serum glucose concentrations were independently associated with endogenous hyperinsulinism. A calculator for probability prediction of endogenous hyperinsulinism was developed including these variables and sex (AUC = 0.94). Older age, female sex, lower body mass index, and lower minimum point-of-care glucose during the first 24 h of fasting were independently associated with serum glucose <55 mg/dL after the first 24 h of fasting. A calculator for predicting probability of serum glucose <55 mg/dL after the first 24 h of fasting was developed including these variables (AUC = 0.84).

**Conclusions:**

Pre- and intra-test variables can identify individuals at low risk for endogenous hyperinsulinism, in whom shortening the 72-hour fasting test could be considered.

## Introduction

In contrast to patients with diabetes treated with insulin or secretagogues, hypoglycemia is a rare condition in people without diabetes [1]. Evaluation and investigation of hypoglycemia should be limited to those who meet Whipple’s triad [2]: low serum glucose concentrations (as measured by a laboratory test); symptoms consistent with hypoglycemia; and improvement of these symptoms after normalization of blood glucose concentrations [3]. According to current guidelines, for individuals without diabetes with hypoglycemia who present with Whipple’s triad and in whom pharmacologic [2,4,5], hormonal [2,6,7], autoimmune [2,8], or health-related causes [2,9] have been excluded, an etiologic study should be performed to rule out endogenous hyperinsulinism. To do this, it is necessary to reproduce hypoglycemia, reaching serum glucose concentrations <55 mg/dL [2]. In patients without diabetes with fasting hypoglycemia, which is more concerning for the diagnosis of insulinoma than postprandial hypoglycemia [2,10,11], the most accepted way to reproduce hypoglycemia <55 mg/dL is to perform a 72-hour fasting test in an inpatient setting [2]. In such a situation of marked hypoglycemia, an individual without endogenous hyperinsulinism should have suppressed insulin secretion [2]. Instead, a person with endogenous hyperinsulinism would maintain elevated endogenous insulin secretion, defined in this context as elevated serum concentrations of both insulin and C-peptide [2]. The presence of endogenous hyperinsulinism in the absence of serum secretagogue metabolites and insulin antibodies should raise the suspicion of insulinoma [2].

The fasting test requires 72 h of fasting unless symptomatic serum hypoglycemia <55 mg/dL or asymptomatic hypoglycemia ≤45 mg/dL occurs previously [2]. During the fasting test, periodic point-of-care blood glucose monitoring is recommended to track the glycemia trajectory [12]. The 72-hour fasting test can be uncomfortable because, in addition to being forced to maintain a prolonged fast, patients might develop unpleasant hypoglycemia symptoms. For this reason, previous studies have proposed shorter fasting tests and concluded that insulinomas can be diagnosed in the first 24 [13] or 48 h of fasting [14,15]. However, other studies have shown that up to 6 %-8% of insulinomas are diagnosed between 48 and 72 h after fasting [16,17]. Hence, the 72-hour fasting test remains the standard in recent reviews and studies [2,12,18,19].

Although a shorter fasting test might not be applicable for all patients, in the era of personalized medicine [20] there could be patients at low risk of endogenous hyperinsulinism for whom shorter fasting tests could be considered, reserving the 72-hour test for high-risk patients. Some pre-test or intra-test variables (such as the analysis of point-of-care glucose trajectory values during the first hours of the fasting test) could allow for the identification of patients at low or high risk of endogenous hyperinsulinism. To our knowledge, no previous studies have identified which variables are associated with a higher risk of endogenous hyperinsulinism. The aim of the present study was to identify pre- and intra-fasting test variables that would allow us to identify patients at low risk of endogenous hyperinsulinism in whom shorter fasting tests could be considered, which would benefit the patient and the healthcare system by reducing these patients’ hospital stay.

## Methods

### Study design and setting

This multicenter retrospective study was performed in patients without diabetes who underwent 72-hour fasting tests between January 2015 and February 2024 in 3 hospitals in northern Spain (2 university hospitals and 1 regional hospital) with the aim of excluding hypoglycemia due to endogenous hyperinsulinism. The main objective of the study was to compare the pre-test characteristics of individuals with and without endogenous hyperinsulinism, as well as the trajectories of their point-of-care glucose during the fasting test. All individuals included in the study reported hypoglycemia (<70 mg/dL) at home (measured by a point-of-care glucometer) or had hypoglycemia detected in the healthcare setting (by laboratory serum glucose or a point-of-care glucometer). None of the individuals included in the study were found to have underlying causes that could explain the hypoglycemia before the fasting test [2,[4], [5], [6], [7], [8], [9]]. Thus, we excluded patients who had an acute illness during the fasting test (hospitalized for other reasons in departments other than endocrinology; n = 2) [2,9], patients for whom the test was performed despite evidence of cortisol deficiency (serum cortisol < 3 mcg/dL in the absence of active corticosteroid treatment; n = 2) or growth hormone deficiency (serum insulin-like growth factor-1 < 84 ng/mL in the context of documented pituitary disease; n = 1) [2,6,7,21], and patients with severe malnutrition (n = 1) [2,9,22]. Given that the administration of exogenous insulin would significantly alter the point-of-care glucose trajectory during the test, we also excluded patients in whom hypoglycemia was found to be related to exogenous insulin administration (either because the patients were found to be self-injecting insulin during the test or because hypoglycemia was found with elevated insulin concentrations and suppressed C-peptide concentrations; n = 3) [2,12,18]. Patients who were unable to complete the 72-hour fasting test despite not reaching serum glucose < 55 mg/dL (n = 3) were also excluded because they could not be diagnosed (i.e., endogenous hyperinsulinism could not be confirmed or excluded). We also excluded 1 patient for whom the fasting test was stopped due to a medical error, despite not having reached serum glucose <55 mg/dL. A total of 64 patients (46 women, median age 45 years, range 16–89 years) met the criteria and were included in the study. Serum secretagogue metabolites [2] or insulin antibodies [8] were not detected in any of these patients, and none of them had an estimated glomerular filtration rate ≤45 mL/min/1.72 m^2^ (i.e., kidney impairment) or serum alanine transaminase >60 U/L (i.e., liver impairment) [2,9]. None of the patients were taking medications known to cause hypoglycemia with high, moderate, or low quality of evidence [2].

All the patients were hospitalized in an endocrinology department to undergo the 72-hour fasting test, and a standardized protocol was followed. The fasting test began 2 h after the start of the last food intake. During the fasting period, patients were allowed to drink only non-caloric, caffeine-free beverages (such as water or infusions), and non-essential medications were discontinued [2]. Patients were allowed to be active during the waking hours [2]. Periodic point-of-care glycemic determinations were performed [12]: initially every 2–6 h, and when point-of-care glucose fell below 55 mg/dL, every 1–2 h. Serum glucose was measured if the point-of-care glucose value was ≤45 mg/dL or if the patient had symptoms of hypoglycemia and the point-of-care glucose value was 46–54 mg/dL. Serum glucose was measured at the end of the test in 15 of the 21 patients who did not reach a point-of-care glucose <55 mg/dL. Testing was stopped before 72 h if symptomatic serum glucose <55 mg/dL or asymptomatic serum glucose ≤45 mg/dL was reached [2]. In all patients with serum glucose <55 mg/dL, serum insulin and C-peptide concentrations were measured in the same hypoglycemic blood sample.

### Ethical issues

The study was approved by the Research Ethics Committee of the Principality of Asturias (code 2023.335), which waived the requirement for informed consent from the study participants, in agreement with Spanish regulations for retrospective studies of clinical records.

### Determinations

#### Outcomes

##### Pre-test probability for a diagnosis of endogenous hyperinsulinism

We aimed to investigate the probability of diagnosing endogenous hyperinsulinism based on pre-test variables available before the start of the fasting test. Endogenous hyperinsulinism was diagnosed in patients whose serum analysis during the fasting test showed serum glucose <55 mg/dL, insulin ≥3.0 μU/mL, and C-peptide ≥0.6 ng/mL [2]. The remaining tests were considered negative for endogenous hyperinsulinism, including for the 6 individuals in whom serum blood samples were not collected because they did not reach point-of-care glucose <55 mg/dL during the 72-hour fast. Serum concentrations of beta-hydroxybutyrate (mg/dL) and proinsulin (pmol/L) were also measured during the fasting test, although unlike insulin and C-peptide, these data were not available for all patients who reached serum glucose <55 mg/dL.

##### Intra-test probability for reaching serum glucose < 55 mg/dL after the first 24 h of fasting

Reaching serum glucose <55 mg/dL is a requirement for the diagnosis of endogenous hyperinsulinism [2]. For individuals whose tests lasted >24 h, we aimed to investigate the probability of reaching serum glucose <55 mg/dL after the first 24 h of fasting based on variables available at hour 24 of the fasting test.

#### Pre-test covariates

##### Demographic factors

Sex and age at the time of the fasting test.

##### Metabolic factors

Body mass index (BMI; calculated from body weight on admission or, if unavailable, the most recent body weight measurement before admission), lowest laboratory serum glucose concentration before admission (not necessarily fasting, but excluding hypoglycemia documented during an oral glucose tolerance test), and glycated hemoglobin (HbA1c) level (on admission or, if unavaiable, the most recent value before admission).

##### Whipple’s triad

A patient was considered to present Whipple’s triad if, prior to the fasting test, he/she had a laboratory serum glucose measurement <70 mg/dL, reported the presence of typical symptoms of hypoglycemia [23], and reported improvement of these symptoms after normalization of blood glucose concentrations [3]. Alternatively, a patient was considered to meet the “point-of-care Whipple’s triad” if, prior to the fasting test, he/she reported any point-of-care glucose measurement <70 mg/dL, reported the presence of typical symptoms of hypoglycemia [23], and reported improvement of these symptoms after normalization of blood glucose concentrations.

##### Characteristics of hypoglycemia prior to admission

A patient was considered to have fasting hypoglycemia before the fasting test if he/she had a previous serum analysis that demonstrated fasting hypoglycemia (<70 mg/dL) or reported that hypoglycemic episodes occurred more than 4 h after ingestion. A patient was considered to have postprandial hypoglycemia before the fasting test if he/she reported that hypoglycemic episodes occurred up to 4 h after ingestion [10].

##### Imaging studies

We recorded whether the patients had undergone an abdominal imaging study (computed tomography, magnetic resonance, or ultrasound) in the 36 months prior to the fasting test and whether such a test showed any type of morphological alteration in the pancreas.

#### Intra-test covariates

All the point-of-care glucose values obtained during the fasting tests were recorded, with each value assigned to the time of the test at which it was obtained. The descending slope of the point-of-care glucose trajectory values during the fasting test was calculated.

### Statistical analyses

Continuous variables were expressed as medians and interquartile ranges. Categorical variables were reported as absolute frequencies and relative frequencies (percentages). Group differences for continuous variables were assessed with the Mann–Whitney *U* test. Chi-squared tests were employed to evaluate group differences for categorical variables. Spearman’s rank test was used to evaluate correlation.

A multivariate logistic regression model was constructed to identify factors associated with endogenous hyperinsulinism and with the probability of reaching serum glucose <55 mg/dL after the first 24 h of fasting. Age and sex were included in the final models alongside representative covariates. Risk calculators were developed using age, sex, and simple and objective (numeric) covariates as predictor variables. HbA1c and history of prior abdominal imaging were not included in the prediction calculators because these variables were not available for a significant proportion of patients in the study. To predict serum glucose levels dropping below 55 mg/dL after the first 24 h of fasting, we used information from point-of-care glucose trajectories during the initial 24 h. Specifically, we considered the lowest point-of-care glucose measurement and the slope of the glucose trajectory on the first day. These slopes were calculated by fitting a linear regression model to each patient’s glucose trajectory. The models’ discriminatory ability was evaluated using the area under the receiver operating characteristic curve (AUC). Bootstrap resampling with 1000 replicates was employed to adjust for potential overfitting and to obtain a more accurate estimate of the AUC.

Additionally, model calibration was assessed graphically and by employing the Brier score. We used hierarchical mixed models to model longitudinal glucose concentration data. The initial model examined the effect of time and endogenous hyperinsulinism on point-of-care glucose values.

The statistical analyses were conducted with the open-source software R, employing the *ggplot2* [24], *pROC* [25], *lme4* [26], and *gamm4* [27] packages.

## Results

### Characteristics and results of 72-hour fasting test serum determinations

Ten (15.6 %) patients had a result consistent with endogenous hyperinsulinism. Once the diagnosis of endogenous hyperinsulinism was established, all 10 patients underwent abdominal imaging to search for pancreatic insulinoma. Supplementary File 1 shows the imaging studies performed on each patient with endogenous hyperinsulinism; functional testing [28] was not performed in any patient. Defined nodular tumors ≥1 cm were found in 6 patients. In 3 of these 6 patients, surgical treatment was performed, confirming the diagnosis of neuroendocrine tumor compatible with insulinoma. In the remaining 3 patients with identified nodular tumors, medical treatment (diazoxide with or without corticosteroids) was chosen due to their age and comorbidities (n = 2), or because the patient refused surgical treatment (n = 1). In 4 patients, no defined tumors were found in the pancreas. Two of these 4 patients are related; thus, a diagnosis of familial adult nesidioblastosis is suspected, although there was no clear overexpression of somatostatin receptors in the nuclear medicine studies performed. These 2 patients continue to have pronounced hypoglycemia requiring treatment with diazoxide while the possibility of partial or total pancreatectomy is being evaluated. Of the remaining 2 patients without identified pancreatic tumors, in one, endogenous hyperinsulinism was probably transient (the patient [number 7 in Supplementary File 1] had no more severe hypoglycemic episodes after a change in his anticonvulsant medication), whereas the other patient was lost to follow-up 1 year after the fasting test was performed.

Table 1A compares the characteristics of the 72-hour fasting tests in those with a result consistent with endogenous hyperinsulinism (serum glucose <55 mg/dL, insulin ≥3.0 μU/mL, and C-peptide ≥0.6 ng/mL) with those without it. Compared with fasting tests performed in individuals without endogenous hyperinsulinism, fasting tests in patients with endogenous hyperinsulinism were shorter in duration and reached lower glucose levels (both serum and point-of-care values). Patients with endogenous hyperinsulinism also showed steeper descending trajectory slopes in point-of-care glucose during the first 24 h of the test and for the entire duration of the test. Serum β-hydroxybutyrate concentrations were significantly lower in patients with endogenous hyperinsulinism. Serum proinsulin concentrations were significantly higher in patients with endogenous hyperinsulinism (Table 1A).

**Table 1:** (A) Characteristics and results of serum determinations of 72-hour fasting tests according to the diagnosis of endogenous hyperinsulinism. (B) Pre-fasting test variables according to the diagnosis of endogenous hyperinsulinism.

**(A) Characteristics and results of serum determinations of 72-hour fasting tests**			
	No endogenous hyperinsulinism (n = 54)	Endogenous hyperinsulinism (n = 10)	P-value
**Test duration** (hours)	72 (72–72)	16 (5–25)	<0.001
**Lowest point-of-care glucose value** (mg/dL)	51 (45–60)	36 (31–40)	<0.001
**Lowest serum glucose concentration** (mg/dL)^1^	53 (47–59)	37 (34–40)	<0.001
**Slope of the trajectory of the point-of-care glucose values during the first 24 h of fasting** ([mg/dL]/h)	−0.92 (−1.26, −0.46)	−1.66 (−4.89, −1.50)	<0.001
**Slope of the trajectory of the point-of-care glucose values during whole fasting test duration** ([mg/dL]/h)	−0.56 (−0.69, −0.43)	−1.66 (−4.89, −1.37)	<0.001
**Serum insulin** (μU/mL)^2^	1.4 (1.0–2.0)	20.2 (4.2–26.4)	<0.001
**Serum C-peptide** (ng/mL)^3^	0.4 (0.4–0.6)	2.9 (1.6–5.5)	<0.001
**Serum β-hydroxybutyrate** (mg/dL)^4^	40.4 (23.1–47.8)	3.5 (1.0–10.3)	<0.001
**Serum pro-insulin** (pmol/L)^5^	0.6 (0.5–1.1)	9.5 (1.3–17.3)	<0.001

**(B) Pre-fasting test variables**			

	No endogenous hyperinsulinism (n = 54)	Endogenous hyperinsulinism (n = 10)	P-value

**Sex** (man)	13 (24.1)	5 (50.0)	0.094
**Age** (years)	43 (40–53)	62 (48–83)	0.007
**Body mass index** (kg/m^2^)^6^	24.5 (21.4–29.2)	30.8 (25.0–34.9)	0.022
**Lowest previous serum glucose concentration** (mg/dL)	79 (66–86)	45 (34–51)	<0.001
**Previous serum hypoglycemia** (yes)	16 (29.6)	10 (100.0)	<0.001
**Previous point-of-care hypoglycemia** (yes)	52 (96.3)	10 (100.0)	0.536
**Glycated hemoglobin** (%)^7^	5.3 (5.0–5.6)	5.0 (4.4–5.1)	0.024
**Whipple’s triad** (yes)^8^	10 (18.5)	8 (80.0)	<0.001
**“Point-of-care Whipple’s triad”** (yes)^9^	42 (77.8)	8 (80.0)	0.876
**Fasting hypoglycemia** (yes)^10^	22 (40.7)	9 (90.0)	0.004
**Postprandial hypoglycemia** (yes)^11^	48 (88.9)	6 (60.0)	0.021
**Pancreatic alteration in imaging studies** (yes)^12^	3 (11.5)	3 (75.0)	0.003

Data are expressed as median and interquartile range (within parentheses) or as absolute numbers and percentage (within parentheses).

1 Data available for 58 patients.

2 Data available for 48 patients.

3 Data available for 51 patients.

4 Data available for 44 patients.

5 Data available for 45 patients.

6 Data available for 63 patients.

7 Data available for 49 patients.

8 A patient was considered to present the Whipple’s triad if he/she had a laboratory serum glucose measurement < 70 mg/dL, reported the presence of typical symptoms of hypoglycemia, and reported improvement of these symptoms after normalization of blood glucose concentrations.

9 A patient was considered to present the “point-of-care Whipple’s triad” if he/she reported any point-of-care glucose measurement < 70 mg/dL, reported the presence of typical symptoms of hypoglycemia, and reported improvement of these symptoms after normalization of blood glucose concentrations.

10 Hypoglycemia was considered as “fasting” if the patient reported that it occurred more than 4 h after ingestion or if it was detected in a fasting serum analysis.

11 Hypoglycemia was considered as “postprandial” if the patient reported that it occurred up to 4 h after ingestion.

12 Data available for 30 patients, as 34 patients had not undergone an imaging study in the 36 months prior to the fasting test.

**Fig. 1:**
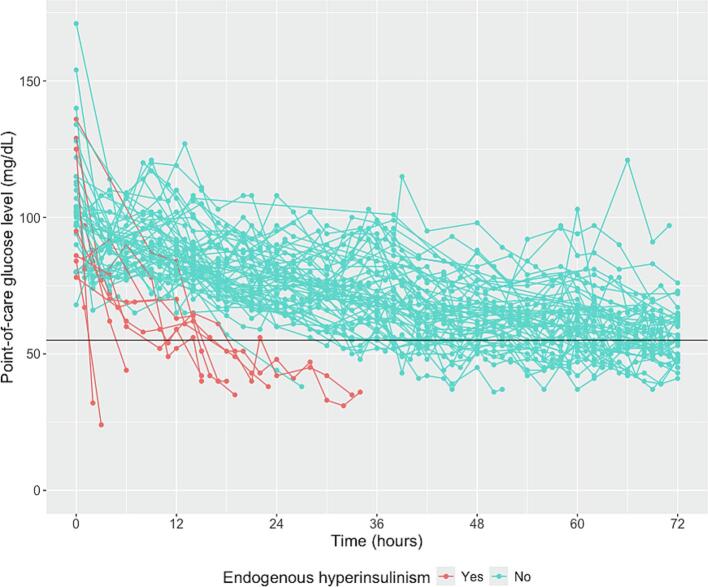
Fasting test point-of-care glucose trajectories of individuals diagnosed with (in red) and without (in blue) endogenous hyperinsulinism. (For interpretation of the references to colour in this figure legend, the reader is referred to the web version of this article.)

Fig. 1 shows the trajectories of the point-of-care glucose tests for the 64 individuals in the study. Supplementary File 2 further shows the point-of-care glucose trajectories for each individual, with the overall glucose trend estimated using a mixed model. These show that the tests of the patients with endogenous hyperinsulinism were shorter in duration, had a steeper descending slope, and reached lower point-of-care glucose values than most of the tests of the individuals without endogenous hyperinsulinism.

### Probability of endogenous hyperinsulinism according to pre-fasting test variables

Table 1B shows the pre-fasting test factors associated with the presence of endogenous hyperinsulinism. Compared with individuals without endogenous hyperinsulinism, patients with endogenous hyperinsulinism had older age, higher body mass index, lower previous minimum serum glucose concentration, a higher frequency of serum hypoglycemia (<70 mg/dL) in previous tests, lower HbA1c levels, more frequent history of the complete Whipple’s triad, a higher frequency of fasting hypoglycemia, less postprandial hypoglycemia, and a higher frequency of pancreatic alteration in imaging studies. Sex and a history of point-of-care hypoglycemia (<70 mg/dL; including the presence of “point-of-care Whipple’s triad”) were not associated with endogenous hyperinsulinism (Table 1B).

**Fig. 2:**
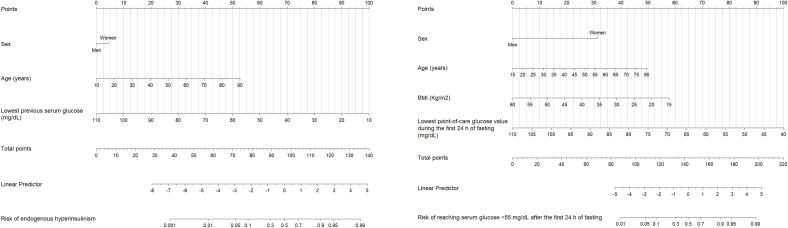
Nomograms for the pre-test prediction of endogenous hyperinsulinism (left panel) and probability of reaching serum glucose <55 mg/dL after the first 24 h of fasting (right panel). Locate the factors on the respective axis and draw a line straight up to the points axis. Add the points for each of the factors and locate the final sum on the total points axis. Draw a line straight down to find the probabilities.

Table 2 shows a multivariate model (logistic regression) of pre-fasting test variables for prediction of endogenous hyperinsulinism before the test. After adjusting for age and sex, the lowest previous serum glucose concentration, a history of Whipple’s triad, HbA1c, and the presence of pancreatic alterations in imaging studies maintained an association with the risk of endogenous hyperinsulinism (Table 2). Based on a model including age (odds ratio [OR] 1.08, 95 % CI 1.01–1.16, p = 0.031), sex (male OR 0.60, 95 % CI 0.06–5.88, p = 0.651), and lowest previous serum glucose concentration (OR 0.89, 95 % CI 0.83–0.96, p = 0.001), we developed a probability calculator to estimate the pre-test probability of endogenous hyperinsulinism (Fig. 2, left panel; Supplementary File 3, Model 1). The model performed very well in discriminating cases from controls, with an AUC of 0.94 (95 % CI 0.88–1.00; bootstrap corrected AUC 0.91). The model showed good agreement between predicted and observed probabilities, with a Brier score of 0.058.

**Table 2:** Multivariate analyses of pre-test variables for prediction of endogenous hyperinsulinism after the fasting test (logistic regression).

	Crude analyses		Age and sex-adjusted analyses	
	OR (95 % CI)	P-value	OR (95 % CI)	P-value
**Age** (years)	1.06 (1.01–1.11)	0.008	1.06 (1.01–1.11)	0.012
**Sex** (man)	3.15 (0.78–12.6)	0.105	2.96 (0.64–13.6)	0.163
**Body mass index** (kg/m^2^)^1^	1.07 (0.98–1.16)	0.121	1.05 (0.95–1.17)	0.318
**Lowest previous serum glucose concentration** (mg/dL)	0.89 (0.84–0.95)	<0.001	0.89 (0.83–0.96)	0.001
**Glycated hemoglobin** (%)^2^	0.08 (0.01–0.70)	0.022	0.01 (0.00–0.32)	0.007
**Fasting hypoglycemia** (yes)^3^	13.0 (1.54–110.8)	0.018	8.83 (0.97–79.7)	0.052
**Whipple’s triad** (yes)^4^	17.6 (3.23–95.8)	<0.001	112.7 (5.18–2453.4)	0.002
**Pancreatic alteration in imaging studies** (yes)^5^	23.0 (1.77–298.4)	0.016	41.2 (1.65–1028.4)	0.023

1 Data available for 63 patients.

2 Data available for 49 patients.

3 Hypoglycemia was considered as “fasting” if the patient reported that it occurred more than 4 h after ingestion or if it was detected in a fasting serum analysis.

4 A patient was considered to present the Whipple’s triad if he/she had a laboratory serum glucose measurement <70 mg/dL, reported the presence of typical symptoms of hypoglycemia, and reported improvement of these symptoms after normalization of blood glucose concentrations.

5 Data available for 30 patients, as 34 patients had not undergone an imaging study in the 36 months prior to the fasting test.

There was a positive correlation between the lowest serum glucose concentration before the fasting test and the minimum point-of-care glucose value reached during the test (Rho = 0.478, p < 0.001), as well as the serum glucose concentration reached during the test (Rho = 0.405, p = 0.002).

### Probability of reaching serum glucose < 55 mg/dL according to pre- and intra-fasting test variables after the first 24 h of fasting

The fasting test finished before 24 h in 8 patients; thus, 56 patients had tests lasting >24 h (Fig. 1). All of the 8 the tests that lasted <24 h had a result compatible with endogenous hyperinsulinism. Thus, the majority (54/56, 96.4 %) of tests lasting >24 h had results that excluded endogenous hyperinsulinism.

Patients with >24-hour tests (n = 56) who reached serum glucose concentrations of <55 mg/dL were more likely to be female, to have lower BMI, and to have lower HbA1c levels than patients who did not reach these glucose concentrations (Table 3). In addition, patients with >24-hour tests who reached serum glucose <55 mg/dL had had a more rapid decline in point-of-care glucose during the first 24 h of testing (i.e., steeper descending slope) and had also reached a lower minimum point-of-care glucose value during the first 24 h of testing (Table 3).

**Table 3:** Pre- and intra-fasting test variables according to the presence of serum glucose < 55 mg/dL after the first 24 h of fasting.

	Did not reach serum glucose <55 mg/dL after the first 24 h of fasting (n = 26)	Reached serum glucose <55 mg/dL after the first 24 h of fasting (n = 30)	P-value
**Pre-test variables**			
**Sex** (man)	10 (38.5)	3 (10.0)	0.012
**Age** (years)	43 (35–53)	44 (40–56)	0.454
**Body mass index** (kg/m^2^)^1^	27.2 (21.9–33.1)	24.0 (20.3–26.2)	0.038
**Lowest previous serum glucose concentration** (mg/dL)	78 (64–90)	79 (66–83)	0.336
**Previous serum hypoglycemia** (yes)	8 (30.8)	10 (33.3)	0.838
**Previous point-of-care hypoglycemia** (yes)	25 (96.2)	29 (96.7)	0.918
**Glycated hemoglobin** (%)^2^	5.5 (5.2–5.7)	5.1 (4.7–5.5)	0.013
**Whipple’s triad** (yes)^3^	6 (23.1)	6 (20.0)	0.780
**“Point-of-care Whipple’s triad”** (yes)^4^	22 (84.6)	22 (73.3)	0.305
**Fasting hypoglycemia** (yes)^5^	10 (38.5)	14 (46.7)	0.536
**Postprandial hypoglycemia** (yes)^6^	22 (84.6)	26 (86.7)	0.827
**Pancreatic alteration in imaging studies** (yes)^7^	3 (23.1)	1 (6.7)	0.216

**Intra-test variables**			
**Lowest point-of-care glucose value during the first 24 h of fasting** (mg/dL)	77 (69–83)	69 (66–76)	0.011
**Slope of the trajectory of the point-of-care glucose values during the first 24 h of fasting** ([mg/dL]/h)	−0.75 (−1.07; −0.35)	−1.14 (−1.54; −0.55)	0.049

Patients whose fasting test lasted less than 24 h (n = 8) were not included. Data are expressed as median and interquartile range (within parentheses) or as absolute numbers and percentage (within parentheses).

1 Data available for 55 patients.

2 Data available for 43 patients.

3 A patient was considered to present the Whipple’s triad if he/she had a laboratory serum glucose measurement <70 mg/dL, reported the presence of typical symptoms of hypoglycemia, and reported improvement of these symptoms after normalization of blood glucose concentrations.

4 A patient was considered to present the “point-of-care Whipple’s triad” if he/she reported any point-of-care glucose measurement <70 mg/dL, reported the presence of typical symptoms of hypoglycemia, and reported improvement of these symptoms after normalization of blood glucose concentrations.

5 Hypoglycemia was considered as “fasting” if the patient reported that it occurred more than 4 h after ingestion or if it was detected in a fasting serum analysis.

6 Hypoglycemia was considered as “postprandial” if the patient reported that it occurred up to 4 h after ingestion.

7 Data available for 28 patients, as the other 28 patients had not undergone an imaging study in the 36 months prior to the fasting test.

Table 4 shows a multivariate model (logistic regression) of pre- and intra-fasting test variables according to the probability of reaching serum glucose <55 mg/dL after the first 24 h of the fasting test (in patients whose tests lasted >24 h). After adjusting for age and sex, female sex, low BMI, and lower minimum point-of-care glucose during the first 24 h of fasting had a positive association with the probability of reaching a serum glucose concentration <55 mg/dL after the first 24 h of fasting (Table 4). Based on a model including age (OR 1.07, 95 % CI 1.00–1.14, p = 0.043), sex (female OR 14.2, 95 % CI 2.04–98.8, p = 0.007), BMI (OR 0.90, 95 % CI 0.81–0.99, p = 0.042), and lowest point-of-care glucose value during the first 24 h of fasting (OR 0.89, 95 % CI 0.81–0.98, p = 0.011), we developed a probability calculator to estimate the intra-test probability of reaching a serum glucose concentration < 55 mg/dL after the first 24 h of fasting (Fig. 2, right panel; Supplementary File 3, Model 2). The model performed very well in discriminating cases from controls, with an AUC of 0.84 (95 % CI 0.73–0.95; bootstrap corrected AUC 0.79). The model showed good agreement between predicted and observed probabilities, with a Brier score of 0.157. According to the calculator, the 2 individuals who had > 24-hour tests and a diagnosis of endogenous hyperinsulinism had, at 24 h after testing, a probability of serum hypoglycemia <55 mg/dL of 98.8 % and 99.7 %, respectively.

**Table 4:** Multivariate analyses of pre- and intra-fasting test variables according to the presence of serum glucose < 55 mg/dL after the first 24 h of fasting (logistic regression).

	Crude analyses		Age and sex-adjusted analyses	
	OR (95 % CI)	P-value	OR (95 % CI)	P-value
**Age** (years)	1.01 (0.97–1.06)	0.442	1.01 (0.97–1.06)	0.489
**Sex** (man)	0.17 (0.04–0.74)	0.018	0.17 (0.04–0.75)	0.019
**Body mass index** (kg/m^2^)^1^	0.91 (0.83–1.00)	0.062	0.89 (0.80–0.99)	0.035
**Slope of the trajectory of the point-of-care glucose values during the first 24 h of fasting** ([mg/dL]/h)	0.45 (0.18–1.10)	0.074	0.45 (0.17–1.19)	0.099
**Lowest point-of-care glucose value during the first 24 h of fasting** (mg/dL)	0.92 (0.86–0.98)	0.014	0.89 (0.83–0.97)	0.008

Patients whose fasting test lasted less than 24 h (n = 8) were not included.

1 Data available for 55 patients.

## Discussion

Our study investigated which pre-test variables were associated with a higher frequency of endogenous hyperinsulinism in 72-hour fasting tests. Older age and low pre-test serum glucose levels were independently associated with increased risk of endogenous hyperinsulinism. We also investigated which variables were associated with increased risk of hypoglycemia <55 mg/dL after the first 24 h of fasting. When analyzing the point-of-care glucose trajectory during the first 24 h of fasting, we observed that patients who reached lower point-of-care glucose values during the first 24 h of fasting were more likely to reach serum glucose <55 mg/dL during the remaining 48 h of testing. Other variables that were independently associated with reaching serum glucose <55 mg/dL after the first 24 h of fasting were older age, female sex, and lower BMI. Using these simple variables, we developed 2 calculators: a first calculator that estimates the risk of endogenous hyperinsulinism before the fasting test is indicated; and a second calculator that estimates, 24 h after the start of the 72-hour fasting test, the risk of reaching serum glucose <55 mg/dL in the remaining 48 h of testing (given that serum glucose <55 mg/dL is a requirement for the diagnosis of endogenous hyperinsulinism). To our knowledge, this is the first study to investigate the factors associated with the diagnosis of endogenous hyperinsulinism in fasting tests. Knowing the risk of endogenous hyperinsulinism before the test and 24 h after the start of the test can be useful to indicate the test or to consider shortening its duration.

According to recent studies [29] and recommendations [2], and even to what Allen O. Whipple stated in the 20th century [3], an etiologic study of hypoglycemia, such as the fasting test, should only be indicated in patients who fulfill Whipple’s triad in its totality. Our results support these recommendations, given that the presence of the complete Whipple’s triad increased the risk of endogenous hyperinsulinism by 17-fold. To strictly fulfill the triad, patients should have serum hypoglycemia confirmed in the laboratory. Thus, the presence of point-of-care (capillary) hypoglycemia would not be sufficient to fulfill Whipple’s triad. Our results showed that the presence of Whipple’s triad at the expense of point-of-care hypoglycemia (what we called “point-of-care Whipple’s triad”) was not associated with a higher frequency of endogenous hyperinsulinism. Furthermore, we observed that the lower the laboratory serum glucose levels before the fasting test, the higher the frequency of endogenous hyperinsulinism and the lower the glycemia reached during the test.

It is known that postprandial hypoglycemia is typically benign, reactive to carbohydrate intake [11]. In contrast, fasting hypoglycemia is more concerning for endogenous hyperinsulinism [2,10]. Our results showed that postprandial hypoglycemia, unlike fasting hypoglycemia, was not associated with a higher frequency of endogenous hyperinsulinism. This finding supports the recommendation not to perform a fasting test in patients with exclusively postprandial hypoglycemia, in whom a mixed meal test might be more appropriate [2]. In addition, patients with endogenous hyperinsulinism had lower HbA1c levels (they probably had lower mean blood glucose levels in the 2–3 months before the test), and higher BMI (possibly because of the need to frequently eat carbohydrates to correct hypoglycemia, as well as due to the anabolic effect of insulin itself) [30].

Low pre-test serum glucose levels and older age were two pre-test variables that were independently associated with a higher frequency of endogenous hyperinsulinism. Using these two simple variables and the patient’s sex, we developed a calculator to estimate the pre-test risk of endogenous hyperinsulinism. A low pre-test risk score for endogenous hyperinsulinism according to the calculator should make physicians reconsider the need to perform a fasting test, especially if the patient presents only with postprandial hypoglycemia, if the HbA1c levels are not low, if the BMI is low (or has not increased since hypoglycemia was reported), and if imaging studies showing no pancreatic changes have been performed.

If the decision is made to perform a fasting test, standard protocols establish a fasting test duration of 72-hours [2,12,18,19]. Previous studies have suggested shortening the duration of fasting tests because of the low frequency of insulinomas diagnosed in the last hours of fasting [[13], [14], [15]]. These shorter fasting protocols have not been implemented in clinical practice because some insulinomas have been diagnosed after 48 h of fasting [16,17]. Our study attempted to identify patients with a low probability of hypoglycemia <55 mg/dL (a necessary condition to diagnose endogenous hyperinsulinism by the fasting test) after 24 h of fasting. Identification of patients at low risk for endogenous hyperinsulinism could allow early discontinuation of testing in these individuals, while continuing the fasting test in patients at higher risk.

In our study, 10 patients had endogenous hyperinsulinism and 8 tests lasted less than 24 h (due to serum hypoglycemia ≤45 mg/dL or symptomatic serum hypoglycemia <55 mg/dL occurring on the first day of fasting). All of these 8 short tests had a result compatible with endogenous hyperinsulinism. Thus, 2 diagnoses of endogenous hyperinsulinism were made after the first 24 h of testing (specifically, at hours 33 and 34), and the vast majority (54/56, 96.4 %) of tests lasting >24 h had results that excluded endogenous hyperinsulinism. Of the 56 tests lasting >24 h, 30 reached serum glucose levels <55 mg/dL. We observed that patients with >24-hour tests who reached serum glucose < 55 mg/dL had a faster decline in point-of-care glucose during the first 24 h of testing and also reached lower minimum point-of-care glucose during the first 24 h of testing. In light of these data, we developed a second calculator to estimate the risk of serum hypoglycemia <55 mg/dL 24 h after the start of the fasting test. The predictor variables of this calculator were age, sex, BMI, and the lowest point-of-care glucose reached during the first 24 h, which is an easier parameter to obtain than the descending slope of the point-of-care glucose trajectories in the first 24 h of testing. For this purpose, male sex and high BMI acted as protective factors for the development of hypoglycemia <55 mg/dL. It is known that in healthy individuals (without endogenous hyperinsulinism), as was the case in 96.4 % of patients with fasting tests >24 h in our sample, physiological hypoglycemia can be more common in lean women [29], who usually have a higher insulin sensitivity than men [31] and than people with a high BMI [32].

Our study has limitations that should be acknowledged. Endogenous hyperinsulinism was diagnosed in only 10 participants. The low number of cases can lead to overfitting in the statistical models and reduces the power to detect significant differences. To mitigate the possibility of overfitting, logistic regression techniques, which provide more stable results, were used alongside bootstrapping techniques that show only slight decreases in the corrected AUC estimates regarding the models' discrimination ability. Only 2 patients in our study had a family history of non-diabetic hypoglycemia (and these 2 patients were related), which did not allow us to assess the influence of family history on the risk of endogenous hyperinsulinism. A family history of insulinoma or multiple endocrine neoplasia-1 (MEN-1) syndrome might increase the risk of endogenous hyperinsulinism, especially in young adults and children [33,34]. Therefore, our results, which show that advanced age is a risk factor for endogenous hyperinsulinism in the population older than 16 years, are not generalizable to children and individuals with a family history of insulinoma or MEN-1 syndrome. The study excluded patients with acute and some chronic conditions that can cause hypoglycemia (including hormone deficiency, severe malnutrition, kidney or liver impairment) [2,6,7,9]. Similarly, patients taking medications with a well-known risk of hypoglycemia [2] were not included. However, the influence of other comorbidities and other chronic medications on the risk of endogenous hyperinsulinism was not evaluated. Retrospective data collection could introduce biases [35]. In this regard, the predictive value of Whipple’s triad might be overestimated, because it is likely that the medical records of patients with less severe hypoglycemia were less detailed about the presence or absence of the 3 components of the triad (for instance, if a patient’s medical record did not show that the clinical signs of hypoglycemia improved after normalization of blood glucose concentrations, it was assumed that the patient did not meet Whipple’s triad). For this reason, we decided not to include Whipple’s triad in the predictive calculators and to replace it with an objective parameter, such as the lowest serum glucose concentration before admission. HbA1c and the presence of pancreatic changes in imaging studies before the fasting test were two variables associated with a higher frequency of endogenous hyperinsulinism; however, they were not performed for all patients before the test. Therefore, these variables were not included in the predictive calculators. Other surrogate tests that could potentially be used to terminate the fasting test earlier, such as ketone monitoring [36], were not evaluated because they were not routinely used in the centers where the study was conducted.

## Conclusions

The probability of endogenous hyperinsulinism is very low in people who do not fully meet Whipple’s triad. In addition, to assess the need for a fasting test, our study provides a simple calculator to estimate the risk of endogenous hyperinsulinism based on age, sex, and pre-test serum glucose levels. Other variables not included in the calculator (such as the presence of fasting hypoglycemia, elevated BMI, low HbA1c, pancreatic changes on previous imaging studies, and a family history of insulinoma or MEN-1 syndrome), might also increase the pre-test risk of endogenous hyperinsulinism.

Once the 72-hour fasting test is initiated, individuals who do not reach low point-of-care glucose values in the first 24 h of the fasting test (i.e., their point-of-care glucose trajectories do not show a rapid decline) are unlikely to have serum glucose <55 mg/dL in the remaining 48 h of testing. Another simple calculator, based on age, sex, BMI, and the lowest point-of-care glucose value in the first 24 h of testing, can calculate the risk of hypoglycemia <55 mg/dL after the first 24 h of fasting. Given that the probability of diagnosing endogenous hyperinsulinism after 24 h of fasting is low, shortening the test could be considered in individuals with a low risk of hypoglycemia <55 mg/dL at 24 h from the start of the test. Future studies are needed to validate these results in different settings.

## CRediT authorship contribution statement

**Tomás González-Vidal:** Investigation, Writing – review & editing. **Óscar Lado-Baleato:** Writing – original draft, Visualization, Formal analysis. **Inés Masid:** Investigation. **Carmen Gándara-Gutiérrez:** Investigation. **Gema Martínez-Tamés:** Investigation. **Jessica Ares:** Writing – review & editing. **Carmen Lambert:** Writing – review & editing. **María Riestra-Fernández:** Writing – review & editing, Investigation. **Francisco Gude:** Visualization, Formal analysis. **Elías Delgado:** Writing – review & editing, Supervision. **Edelmiro Menéndez-Torre:** Writing – review & editing, Supervision.

## Funding

ÓLB was supported by ISCIII Support Platforms for Clinical Research (ISCIII/PT23/00118/Co-funded by European Union). This research did not receive any other specific grant from funding agencies in the public, commercial, or not-for-profit sectors.

## Declaration of competing interest

The authors declare that they have no known competing financial interests or personal relationships that could have appeared to influence the work reported in this paper.

## Data Availability

The data that support the findings of this study are available from the corresponding author upon reasonable request. The data are not publicly available due to ethical restrictions.
