# Supplementary material for: Variables associated with endogenous hyperinsulinism in hypoglycemia diagnosis. Could the 72-hour fasting test be shortened in low-risk patients?
Source: J Clin Transl Endocrinol. 2025 Mar 7;40:100386. doi: 10.1016/j.jcte.2025.100386 (PMC11950777; doi:10.1016/j.jcte.2025.100386)
Supplement: Supplementary Data 1 [file mmc1.pdf]

Supplementary Table 1. Abdominal imaging studies in patients with endogenous hyperinsulinism

**Patient number**	**Abdominal imaging studies**				
	**CT**	**MRI**	**Endoscopic ultrasound**	**PET-CT with 18F-DOPA**	**PET-CT with 68GA-DOTA-TOC**
**Patients in whom pancreatic nodular tumors ≥1 cm were found on abdominal imaging studies**					
1					
2					
3					
4					
5					
6					
**Patients in whom defined pancreatic tumors were not found on abdominal imaging studies**					
7					
8					
9					
10					

Green cells indicate that the study was performed and a defined pancreatic tumor was found. Red cells indicate that the study was performed but no defined pancreatic tumor was found. White cells indicate that the study was not performed.

CT, computed tomography. MRI, magnetic resonance imaging. PET, positron emission tomography. 18F-DOPA, fluorine-18-L-dihydroxyphenylalanine. GA68-DOTA-TOC, gallium-68 dodecanetetraacetic acid 0-Phe1-Tyr3 octreotide.
