# Supplementary figures and images for: Variables associated with endogenous hyperinsulinism in hypoglycemia diagnosis. Could the 72-hour fasting test be shortened in low-risk patients?
Source: J Clin Transl Endocrinol. 2025 Mar 7;40:100386. doi: 10.1016/j.jcte.2025.100386 (PMC11950777; doi:10.1016/j.jcte.2025.100386)

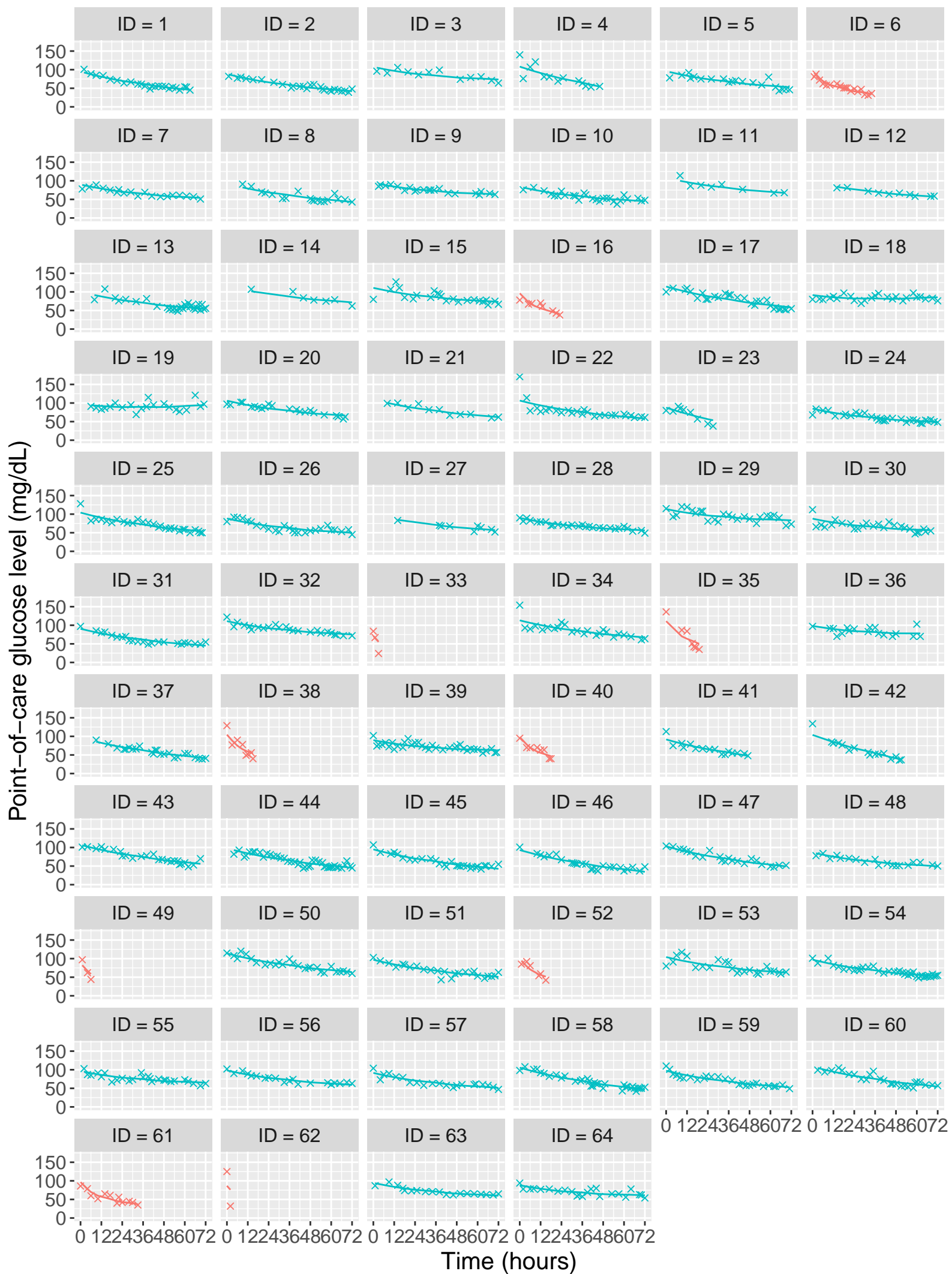

Supplement: Supplementary Data 2 [file mmc2.pdf]
